# An Unusual Cause of a Pain in the Neck: Pseudoaneurysm from Tuberculous Lymphadenitis

**DOI:** 10.1155/2012/391940

**Published:** 2012-08-28

**Authors:** B. Kennedy, D. R. Curran, A. Brady, T. M. O'Connor

**Affiliations:** ^1^Department of Respiratory Medicine, Mercy University Hospital, Grenville Place, Cork, Ireland; ^2^Department of Radiology, Mercy University Hospital, Grenville Place, Cork, Ireland

## Abstract

A pseudoaneurysm is a haematoma which is surrounded by connective tissue and communicates with the lumen of a ruptured blood vessel. It has no true defined capsule. We describe a case of tuberculous pseudoaneurysm. This is a rare complication of tuberculosis. The clinical presentation of these lesions is highly variable. Definitive diagnosis should consist of contrast-enhanced CT and arteriography. Treatment should include repair of the arterial wall by surgery, endovascular stent-graft insertion, or embolization followed by a full course of antituberculous chemotherapy. Our case is highly unusual in that the pseudoaneurysm arose from the subclavian vasculature in a patient with extrapulmonary tuberculosis only.

## 1. Introduction

A pseudoaneurysm is a haematoma which is surrounded by connective tissue and communicates with the lumen of a ruptured blood vessel. It has no true defined capsule. These lesions are a rare complication of tuberculosis. Nevertheless they must be recognised promptly as perforation or rupture may lead to haemodynamic shock and death. Previous case reports predominantly describe pseudoaneurysms of the thoracic or abdominal aorta occurring in patients with pulmonary tuberculosis. Our case is highly unusual in that it describes a pseudoaneurysm of the subclavian vasculature in a patient with extrapulmonary tuberculosis only. 

## 2. Case Report

**Figure 1:**
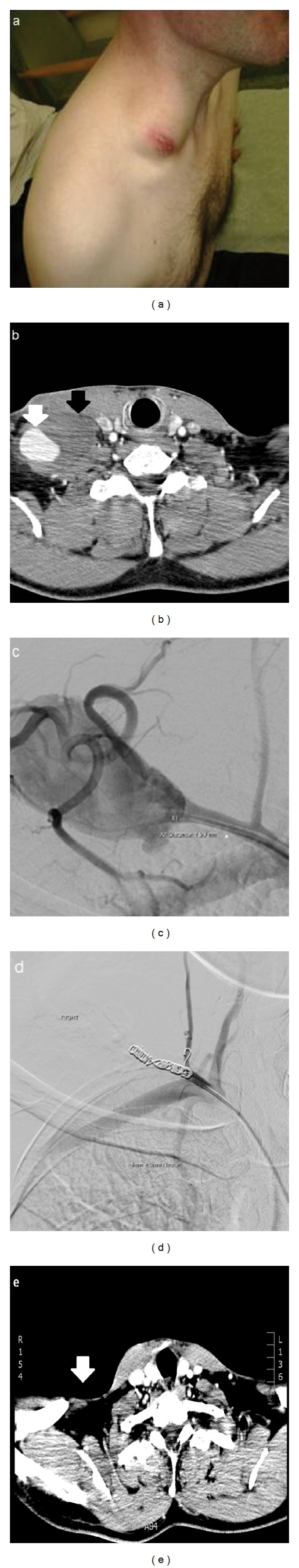
Right supraclavicular mass. (a) Supraclavicular mass at presentation. (b) Supraclavicular mass visualised on CT (black arrow-head) and vascular blush indicating pseudoaneurysm (white arrow-head). (c) Angiogram demonstrating pseudoaneurysm arising from branch of right dorsal scapular artery. (d) Angiogram after embolisation demonstrating occlusion of feeding vessel. (e) CT neck after 6 months of antituberculous therapy demonstrating resolution of supraclavicular mass (white arrow).

A forty-year-old gentleman was referred with a painful right supraclavicular mass. The mass was first noticed two months prior to presentation and had enlarged considerably over this period. There was associated weight loss and malaise. There was no history of fever, cough, or dyspnoea. The patient was a nonsmoker who drank alcohol occasionally. Physical examination revealed a mildly tender, firm, and mobile mass in the right supraclavicular fossa (Figure 1(a)). No pulsation was appreciated. No other lymph nodes were palpable and no other physical abnormalities were detected.

Chest radiograph and routine blood tests were normal. Mantoux test was positive at 19 mm. White caseous material was aspirated from the mass. Microscopy revealed caseating granulomatous lymphadenitis suggestive of tuberculosis (TB). No acid-fast bacilli were seen on smear and no organisms were grown from culture. Screening tests for HIV and Hepatitis B and C were negative. CT neck and thorax were performed to assess for other sites of lymphadenopathy; right upper and lower paratracheal and right hilar nodes were also found to be enlarged. The right supraclavicular mass measured 5.7 × 5.4 cm (Figure 1(b), black arrow-head). It exhibited an intense vascular blush indicating erosion into a blood vessel with pseudoaneurysm formation (Figure 1(b), white arrow-head). Right subclavian angiography showed active extravasation from a branch of the right dorsal scapular artery into the pseudoaneurysm (Figure 1(c)). This artery was selectively cannulated with a 5-French multipurpose catheter and then occluded with two Nestor coils (Figure 1(d)). There was no clinical evidence of right upper limb ischemia before or after detection of the pseudoaneurysm.

The patient was placed on isoniazid, rifampicin, ethambutol, and pyrazinamide. The patient received six months of antituberculous chemotherapy. Three months after treatment was completed, the patient was reassessed. He remained clinically well; there was no evidence of the mass on examination. CT neck was also performed at time of followup. This demonstrated complete resolution of the supraclavicular mass (Figure 1(e), white arrow-head). No residual vascular blush was visible, and the native right subclavian, vertebral, and common carotid arteries remained patent. No further interventions occurred as there was no clinical or radiological evidence of residual disease. 

## 3. Discussion

The formation of a pseudoaneurysm begins with a defect in the arterial wall; this defect may arise from arterial wall inflammation or from trauma (e.g., coronary angiography). Blood then leaks through the defect into the extravascular space where it is encapsulated by connective tissue and becomes organised. Pseudoaneurysms differ from true aneurysms which are defined as a localised dilatation of all layers of the arterial wall. 

Mycotic aneurysm secondary to TB is a rare event. In a study of 22,792 necropsies between 1902 and 1955, Parkhurst reported only a single tuberculous aneurysm [1]. Similarly, Ohta noted only 25 reports of these aneurysm between 1990 and 2000 [2]. The vast majority of tuberculous aneurysms are pseudoaneurysms (87%) but true (9%) or dissecting (4%) aneurysms have been described. They are typically saccular in shape and usually occur as a solitary lesion [3]. In recent times, the most common location of tuberculous aneurysms has been the thoracic aorta [2]. This is due to the anatomical proximity of the thoracic aorta to the lungs and mediastinum where TB most commonly occurs. 

A literature search revealed only two previous reports of tuberculous aneurysms involving the subclavian artery; one of these aneurysms appeared to arise from miliary TB [4] while the other was a consequence of pulmonary TB [5]. In our patient the pseudoaneurysm arose from the erosion of a tuberculous lymph node into a branch of the subclavian artery. The pathogenesis observed in our patient is consistent with a report by Long et al. which found that 75% of tuberculous aneurysms of the aorta arose from a nearby focus of inflammation eroding through the vessel wall. In the other 25% of cases, tuberculous aneurysms likely arose from blood-borne bacilli seeding directly on to the vessel wall or from bacilli carried to the vessel wall by the vasa vasorum [6]. 

Recognition of tuberculous aneurysms remains difficult because of their rarity. This is compounded by the diverse clinical presentation of these aneurysms. They may present as an enlarging mass that is clinically palpable or visible on radiology. Patients may also describe pain at the site of the aneurysm which may be accompanied by fever. Rupture or perforation of the aneurysm may lead to major haemorrhage and haemodynamic shock [3]. Bleeding can also arise from the formation of a fistula between the aneurysm and a nearby organ such as the trachea or intestines which can lead to massive haemoptysis [7] or GI bleeding [8].

It must be borne in mind that tuberculous aneurysms can occur in patients without a prior diagnosis of TB. Long et al. reported that 38% of patients with tuberculous aneurysms of the aorta did not have a diagnosis of TB at the time of presentation [6]. A tuberculous aetiology may be suspected from the clinical presentation or radiological findings [9]. In these patients, TB may be diagnosed definitively by histological examination and/or TB culture of a section of aneurysm wall which is obtained intraoperatively [3]. 

Although unavailable at the time our patient was assessed, we strongly recommend ultrasound for the initial investigation of suspected tuberculous lymphadenopathy; ultrasound facilitates prompt examination at the bed-side, provides visual guidance during aspiration and may also identify abnormal vascular structures such as pseudoaneurysms. Once pseudoaneurysm is suspected, contrast-enhanced CT should be the investigation of choice [3, 6, 9]. MRI may be an alternative to CT [3] and in some cases may provide additional anatomical information [6]. Once the aneurysm has been identified, angiography of the affected blood vessels is indicated if the clinical circumstances permit [3, 6, 9, 10]. 

Treatment of tuberculous aneurysm must include both repair of the vessel wall and antituberculous chemotherapy. Repair of the vessel wall can be performed by surgery [3, 6], endovascular stent-graft insertion [9, 11] or embolization of the affected artery [12]. Until recently, surgery had been the only option for successful repair of the vessel wall. However in recent times, endovascular stent-graft insertion and arterial embolization have emerged as efficacious techniques. The advantage of these techniques over surgery is that they are less invasive; as such they are appropriate in more debilitated patients and should also reduce hospital stay. The disadvantage is that they do not permit extensive excision or debridement of infected tissue which may increase the risk of persistent infection [9, 11]. Thus the optimal treatment strategy should include consideration of the patient's functional status as well as a review of any available imaging; this ensures that the patient is robust enough to survive the procedure and that the risk of residual infection is minimised. Finally the importance of antituberculous chemotherapy cannot be overemphasised; in a review of 39 cases of tuberculous aneurysm of the aorta, none survived without chemotherapy [6]. 

## 4. Conclusion

In summary, we report a case of a forty-year-old gentleman with tuberculous lymphadenitis with pseudoaneurysm formation. Pseudoaneurysm is a rare complication of TB. Its clinical presentation is variable making diagnosis difficult. It is recommended that contrast-enhanced CT is the investigation of choice. This should be followed by angiography. Treatment should include repair of the arterial wall by surgery, endovascular stent-graft insertion, or embolization as well as a full course of antituberculous chemotherapy. A high index of suspicion is required to diagnose these aneurysms; if unrecognised, they may rupture, perforate, or fistulate with possibly fatal consequences.
